# Supplementary material for: Robust Beam Selection Based on Water Equivalent Thickness Analysis in Passive Scattering Carbon-Ion Radiotherapy for Pancreatic Cancer
Source: Cancers (Basel). 2023 Apr 28;15(9):2520. doi: 10.3390/cancers15092520 (PMC10177227; doi:10.3390/cancers15092520)
Supplement: Supplementary file 1 [file cancers-15-02520-s001.zip › cancers-2210813-supplementary.pdf]

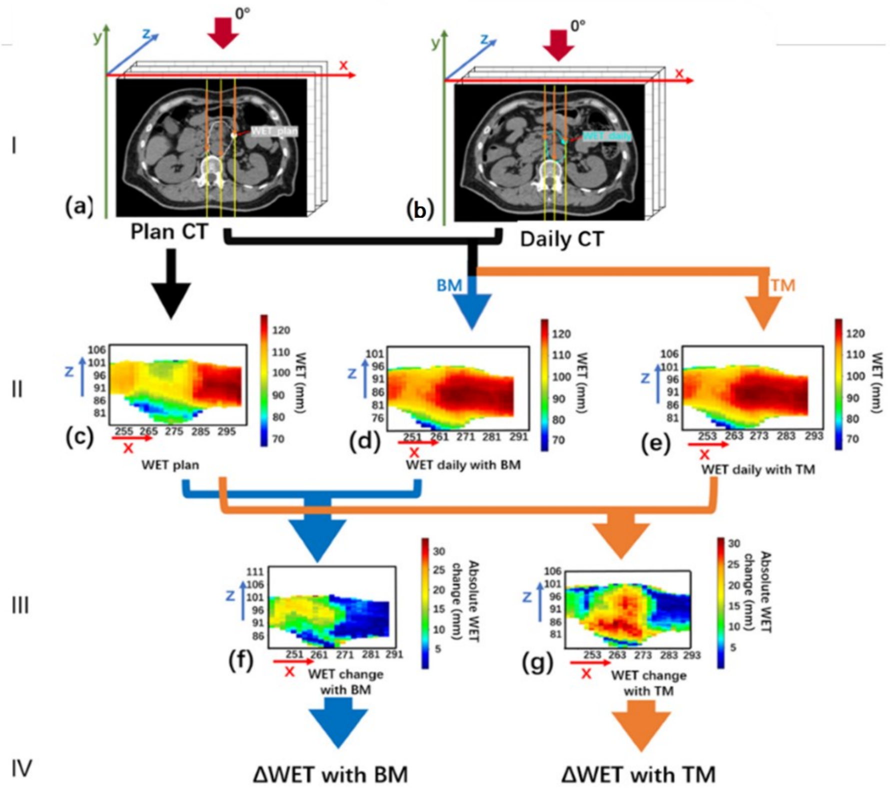

Figure S1. An example of the workflow of the WET changes at 0°. (a) and (b): The CT (plan and daily) images and reference coordinate axes. (c), (d), and (e): 2D matrix of WET_plan, WET_daily with BM, and TM in the 0° heat map, respectively. The coordinate systems for (d) and (e) were adjusted using BM and TM based on the (c). (f) and (g): 2D matrix of the absolute WET change heat map of the overlapping region with BM and TM in 0° beam pass, respectively. ΔWET is the average value of the absolute WET change; red arrows: beam direction. Orange arrows: WET calculation range, yellow line: identified paths. The cross-section of each identified path is equal to the voxel of CT (1.07 mm × 1.07 mm).

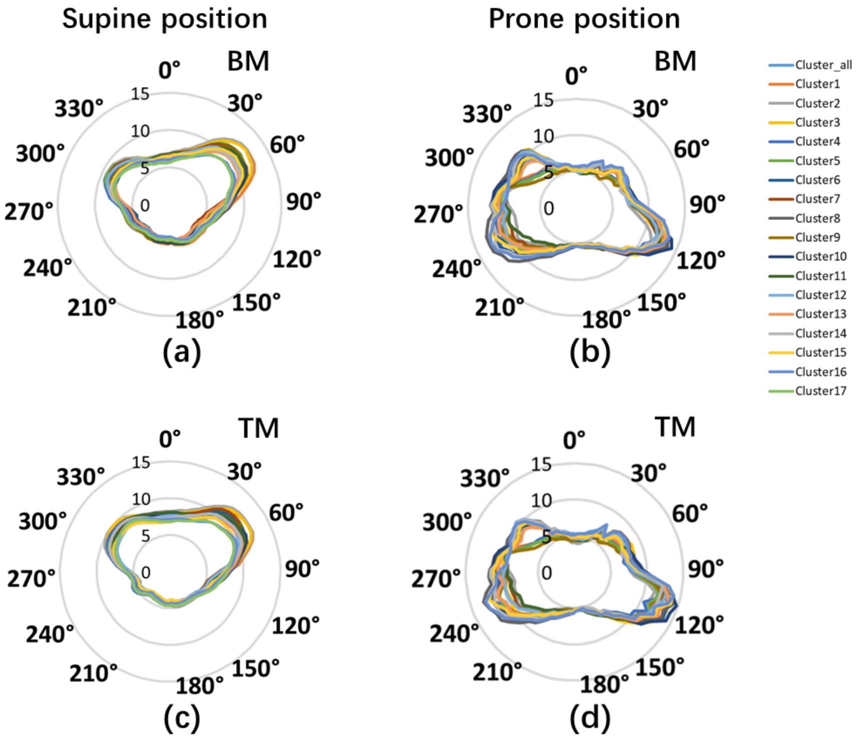

Figure S2. The polar plots show the mean ΔWET for 18 patient clusters, with bone matching (BM) (a-b) and tumor matching (TM) (c-d) shown separately. Each line in the polar plots represents a different cluster of patients, with a total of 18 clusters used including 17 clusters with 6 patients and one cluster (cluster_all) with 8 patients. For the cluster including patient 5, the mean values in prone position were calculated using data from the remaining five patients due to the unavailability of CT data for patient 5 in the prone position.

Table S1. Beam arrangement with three BCs for each fraction

Table S1.1 Beam arrangement in the first nine fractions (for CTV1)

Patient number	position	Beam configuration	The first nine fractions								
			1st	2nd	3rd	4th	5th	6th	7th	8th	9th
1-8	Supine	BC_original (0° ,90° and 270°)	0°	90°	270°	0°	90°	270°	0°	90°	270°
		BC_fixed (355° ,110° and 255°)	355°	110°	255°	355°	110°	255°	355°	110°	255°
		BC_gantry (345° ,150° and 210°)	345°	150°	210°	345°	150°	210°	345°	150°	210°

Table S1.2 Beam arrangement in the last three fractions (for CTV2)

Patient number	position	Beam configuration	The three last fractions		
			10th	11th	12th
1-4,6-8	Prone	BC_original (180°)	180°	180°	180°

		BC_fixed (180°)	180°	180°	180°
		BC_gantry (0°)	0°	0°	0°
		BC_original (0°)	0°	0°	0°
5	Supine	BC_fixed (0°)	0°	0°	0°
		BC_gantry (0°)	0°	0°	0°

Table S2. Three beam schedules for the BC_gantry

Beam schedule												
	**Supine position**									**Prone position**		
day	1st	2nd	3rd	4th	5th	6th	7th	8th	9th	10th	11th	12th
Order 1*	345°	150°	210°	345°	150°	210°	345°	150°	210°	0°	0°	0°
Order 2	150°	150°	150°	210°	210°	210°	345°	345°	345°	0°	0°	0°
Order 3	345°	345°	345°	150°	150°	150°	210°	210°	210°	0°	0°	0°

* Shown in the main text

Table S3. V95% of CTV1 and CTV2 with the BC_gantry in three schedules

		Accumulated dose					
		**TM**			**BM**		
		**Order 1**	**Order 2**	**Order 3**	**Order 1**	**Order 2**	**Order 3**
Case 1	CTV1	72.9%	81.2%	68.1%	79.3%	78.0%	68.0%
	CTV2	77.5%	82.8%	75.2%	76.5%	75.7%	69.6%
Case 2	CTV1	98.1%	97.0%	99.2%	95.7%	95.4%	96.4%
	CTV2	96.3%	96.2%	99.4%	95.7%	96.1%	96.5%
Case 3	CTV1	88.3%	85.1%	92.6%	88.4%	86.4%	90.1%
	CTV2	92.6%	89.2%	91.4%	89.1%	87.2%	89.4%
Case 4	CTV1	94.2%	94.8%	93.7%	94.7%	94.6%	93.7%
	CTV2	93.7%	93.2%	93.4%	93.2%	93.7%	92.6%
Case 5	CTV1	90.3%	87.9%	90.6%	76.9%	73.5%	76.4%
	CTV2	91.8%	87.5%	90.7%	49.8%	48.0%	48.5%
Case 6	CTV1	94.4%	92.8%	92.2%	91.5%	92.2%	91.0%
	CTV2	96.2%	93.6%	97.1%	94.0%	94.7%	94.3%
Case 7	CTV1	93.1%	91.3%	91.1%	86.4%	85.9%	85.5%
	CTV2	94.4%	91.4%	93.3%	91.8%	92.1%	90.5%
Case 8	CTV1	98.6%	99.3%	98.5%	99.1%	99.2%	98.7%
	CTV2	99.8%	100.0%	100.0%	100.0%	99.8%	98.9%

CTV1 is based on the first nine fractions. CTV2 is based on all twelve fractions.
